# Treatment of Hemorrhoids

**Published:** 1878-03-20

**Authors:** L. Q. Lincecum

**Affiliations:** Lampasas, Texas


### SOUTHERN MEDICAL RECORD.

VOL. VIII. ATLANTA, GA., MARCH 20, 1878. NUMBER 3.

EDITORS:

THOMAS S. POWELL, M. D. W. T. GOLDSMITH, M. D. R. C. WORD, M. D.

*R. C. WORD, M. D., Managing Editor.*

SUBSCRIPTION—$2.00 PER ANNUM, IN ADVANCE.

☞ All Communications and Letters on Business connected with THE RECORD, for 1877 and 1878, must be addressed to the Managing Editor.

### *ORIGINAL AND SELECTED ARTICLES.*

### TREATMENT OF HÆMORRHOIDS.

BY L. Q. LINCECUM, OF TEXAS.

I send you some experiments in the treatment of piles and constipation, and some thoughts on diet.

From long experience, tobacco surpasses all other applications in the treatment of piles. If the inflammation and pain be very urgent, it is best to reduce it with an alkaline wash at the beginning; for this purpose I use the caustic potassa, in proportion of one grain of the caustic to two ounces of soft water (or bicarb. soda dr. i. to oz. i. aqua). Sometimes the pile tumors are turned out, and so much inflamed, and so extremely irritable, that the patient cannot bear the touch of anything on them. Use light-corded bats of cotton, wet with the cold alkaline wash, and at first let the wash drip on the tumors from the cotton until the tumors are wet; continue it until the irritation is reduced a little, then lay the wet cotton on the tumor, gently pressing it down with the hand. After a short time, re-wet and re-apply the cotton again and again, until the patient complains of stinging or smarting from the alkalie; then wash it off with the cotton wet in clear, cold water two or three times, after which a bat of cotton, or a soft rag, well wet with an infusion of tobacco, may be laid on the pile tumors, and let it remain ten or fifteen minutes. But if the patient sickens, or complains of any pains, or gripings of the bowels, before the fifteen minutes expire, the tobacco must be washed off with warm water, and an effort made to press the pile back into the bowel, if practicable. The tobacco produces no irritation, but has a soothing effect. With some patients, however, when too freely applied, it nauseates the stomach, and produces severe griping of the bowels. To guard against these unpleasant symptoms, the patient must be watched, and at the first indication of the sick stomach, griping, or giddiness, the tobacco must be removed. If the pile tumors cannot be returned then, wait awhile and apply the tobacco again; it will not require many experiments before the patient will learn how long he can bear it, and how often to apply it. There is no necessity for griping and sickening the patient. If properly managed, it may be applied often in the course of the day without any disagreeable symptoms at all.

After the hemorrhoidal tumors have been returned, the tobacco may be used occasionally in the form of suppositories. Take an ordinary quid of tobacco, wet and soften it with warm water, roll it in a proper form, and then introduce into the anus with the finger. The time for performing this operation should be at the time of going to stool. It will soon produce an action from the bowels.

No person having the piles should ever take purgative medicine. He may so use the tobacco suppositories as to do away with the *supposed* necessity for purging medicines altogether. Should the tobacco suppositories fail to evacuate the bowels, the patient must resort to the use of the warm water enemata. Cathartics are positively inadmissible with pile cases if the intention is to cure them. It must be very obstinate costiveness that tobacco, applied as above directed, cannot relieve. Better wait, eat less, and try the tobacco again, than to drive the blood into the hemorrhoidal vessels with the purges.

When internal piles are throbbing and painful, the tobacco suppository should be applied immediately; it gives instant relief. By prompt application of the tobacco, at the first indication of a returning paroxysm of piles, relief will be certain; and by thus throwing off the paroxysms, from time to time, the parts affected will be gradually restored, until a final cure may be produced. I have known a number of radical cures produced by a careful adherence to the above treatment.

As a general rule, when constipation occurs, as it does sometimes, in apparent good health, the use of the tobacco suppository will obviate the difficulty in a few minutes. The tobacco may be used for this purpose, day by day, without danger of any kind of constitutional injury, and the patient can, by regular and prompt attention to it daily, at a certain hour, induce regular, habitual action of the bowels, and he will find, after having pretty well established this habitual action of the bowels, and it occurs to him that he has gone over the usual hour without an action, that if he will get the tobacco in hand and start to walk, preparing the suppository as he goes, the peristaltic motion of his bowels will immediately commence, and the desire for an action will become pretty pressing without applying the suppository. He should, nevertheless, apply the tobacco to encourage the habit.

The explanation of the action of the will power upon the involuntary nerves, evidently and distinctly manifested in this case, belongs to another department in the investigation of animal motions. Some people are so completely under the animal control, that after devouring a heavy meal of a variety of dishes, they take a purging bolus as soon as they rise from the table, for fear their stomach will not be able to empty itself in time for them to enjoy the next meal appropriately. After the stomach has been forced to pass its contents downwards, the *patient*, having his hopes set upon the next meal, resorts to a quantity of stimulants to restore, if possible, the flattened feeling of the stomach by the return of that longed-for event, the hour for dinner. Dinner comes at last, and he is happily seated at the table, in a select position for the greatest facilities. He smacks his mouth and casts his fierce eyes around, indicative of much firmness and resolution; his lips are drawn to a straight line across his eager visage, and the work of destruction begins. First process is to sharpen appetite, and calling for vinegar, pickles, and catsups, he goes to work cutting and slashing. With him, the dinner table is not a place to discuss politics, or any other subject, except such subjects as are spread before him on the table. To him, the dinner hour is an hour of chewing and swallowing. Then comes what he calls the digestive hour, during which time he lies down to sleep. Instead of his *siesta* being the digestive hour, it is in reality the fermentative hour, and he rises from his lounge, swelled almost to the point of explosion, to relieve which he swallows his cathartic bolus, and so on, cramming and blowing out with his purges, all of which he will freely and pleasantly relate to any one that will listen to him; offering it as his plan, which he heartily recommends to his hearers as the best and most approved plan for eating the greatest quantities. He has tried it, and he ought to know. He will conclude with a smile of self-approbation on his greasy countenance, and he really thinks and believes what he says. Meet him out from home at any time, and enquire for his health, his answer will be: "Well, thank God, I am able to eat my allowance with a tolerable good stomach, but I am never to say well. I am troubled with frequent headaches, colic, and the like; also, I have rheumatism at nights, and piles all the time, most awfully. I cannot imagine what is the matter or cause of all this, for it seems like I eat heartily enough. It may be possible that I do not eat enough. I have been trying lately to find a good medicine that will increase my appetite, thinking, maybe, if I could have a stomach to eat more, that my complaints would leave me." All this, too, expressed in tones of earnestness, and such seeming innocence, that he could not be accused of having the slightest idea of the cause of his said aches, pains and hemorrhoids. "And the Lord smote them with emerods."

In all this, there is not a single word from the intellectual department in his organism; it is from the animal group, and out of the pale of reason. We smile at the character described in the above, and yet there is not one in a hundred of us who does not, to a greater or less extent, represent the said gormandizer. We have all yielded ourselves up to the animal suggestions of the table, until nothing *less* than a painful distention of the stomach will satisfy us. The proof of this statement lies in the fact that you can hardly find a person, male or female, twenty-five years of age, who is not more or less dyspeptic; and I am well assured that if they would confine themselves to one-half the amount they are in the habit of eating, they would have no pains nor aches to complain of, and their mental and physical energies would be so different that they can now form no conception of the change that would follow such a course of dieting.

Nature's operations are noiseless, and when any one feels the slightest degree of flatulency or rumbling in the bowels, he may set it down that he has overcharged the digestive functions, and that to continue the abuse will sooner or later bring on disease of some kind. Most commonly, such impropriety develops itself in the form of dyspepsia, but this energy-destroying disease seldom appears twice in the same garb. It takes on the form of many complaints. They are, however, all eating complaints, and the unfortunate sufferer is always found searching for some kind of food that will suit his case, so that he may eat freely of it without injury. He finds many such articles of food, but in a few days his stomach rejects it too, and for the same reason that has forced him to abandon many other agreeable articles of diet, and that reason was overeating. The rule should be, when he finds a suitable article, to eat very sparingly of it. It takes but little to replenish the system, and every mouthful over that amouut does harm, whether the eater is sick or well. To consume a greater quantity of food than is required to sustain the system at a healthy, cheerful pitch, is not only a weakness, but a positive damage to both mental and physical man.

LAMPASAS, TEXAS.

